# ELISPOTs produced by CD8 and CD4 cells follow log normal size distribution

**DOI:** 10.1186/2051-1426-1-S1-P98

**Published:** 2013-11-07

**Authors:** Jaya Ghosh, Kinga Karacsony, Wenji Zhang, Oleg Targoni, Ioana Moldovan, Paul V Lehmann, Alexey Y Karulin

**Affiliations:** 1 R&D, Cellular Technology Ltd., Shaker Hts., OH, USA

## Introduction

Each positive well in ELISPOT assays always contain spots of variable sizes that range from micrometers up to a millimeter in diameter. Therefore, when it comes to counting ELISPOTs it is critical to decide how to set the lower and the upper spot size thresholds to discriminate between non-specific background noise, spots produced by individual T cells, and spots formed by T cell clusters. If the spot sizes produced by T cells would not follow a certain statistical distribution, the size thresholds (gates) for counting would need to be set based on subjective judgment, thus leading to variability in spot counts when parameters are set by different investigators. In contrast, if the spot sizes follow a defined statistical distribution, precise predictions can be made as to the minimal and maximal spot sizes that belong to a given population. Hence, the gates could be set automatically ensuring counting results that are independent of subjective judgments.

## Methods

We studied the distributional properties of IFN-γ, IL-2, IL-4, IL-5 and IL-17 ELISPOTs elicited in PBMC of 24 healthy donors after stimulation of each donor cells with 32 individual viral peptides representing defined HLA Class I-restricted epitopes for CD8 cells (individual CEF peptides) and with inactivated CMV or EBV virions that recall CD4 cells.

## Results

A total of 334 CD8 and 80 CD4 positive recall responses have been analyzed so far. Invariably, for all donors, antigens, and cytokines, the spot size distributions followed a Log Normal function with significance levels over 5% according to the Kolmogorov-Smirnov test. Coefficient of Variation (CV) of mean Log spot sizes for different donor/antigen combinations in the same experiment was between 4% and 7% (Figures 2, 4 and 5) and much smaller then CV of log sizes for a single experimental distribution ~25% average (Figure 1) resulting in minimal variance in gate positions.

## Conclusions

The data establish that ELISPOTs generated by CD8 or CD4 cells producing IFN-γ, IL-2, IL-4, IL-5, and IL-17 show size distributions that follow a standard known probability function, Log Normal. Therefore, size gates for counting such ELISPOTs can be set automatically by means of statistics permitting harmonization of results obtained by different investigators.

